# A safe and efficient, naturally ventilated structure for COVID-19 surge capacity in Singapore

**DOI:** 10.1017/ice.2020.309

**Published:** 2020-06-24

**Authors:** Natasha Bagdasarian, Ian Mathews, Alexander J. Y. Ng, Eugene H. Liu, Clara Sin, Malcolm Mahadevan, Dale A. Fisher

**Affiliations:** 1 Division of Infectious Diseases, Department of Medicine, National University Hospital, National University Health System, Singapore, Singapore; 2 Department of Medicine, Yong Loo Lin School of Medicine, National University of Singapore, Singapore, Singapore; 3 Emergency Medicine Department, National University Hospital, National University Health System, Singapore, Singapore; 4 Department of Anesthesia, National University Hospital, National University Health System, Singapore, Singapore; 5 Hospital Operations, National University Hospital, National University Health System, Singapore, Singapore

*To the Editor*—The need for airborne infectious isolation (AII) surge capacity is a problem for hospitals globally,^1,2^ and this problem has been highlighted during the COVID-19 pandemic.^3^ Alternatives to AII rooms have been proposed for surge periods. Airborne isolation units can be quickly converted to cohort patients with the same illness, typically requiring negative pressure relative to adjacent areas and adequate ventilation without recirculation to other areas.^2,4^ Mobile containment units have been used in conflict zones^5^ and in outbreak response settings^6,7^ including the current pandemic. Many countries still face spiraling numbers of cases, while others are attempting to return to normalcy after a period of lockdown; some of these countries may experience a second wave even larger than the first. Here, we describe the design and function of a low-cost, naturally ventilated temporary structure to increase EMD capacity during the COVID-19 response in Singapore.

National University Hospital (NUH) is a 1,200-bed tertiary-care academic hospital in Singapore, with an emergency medicine department (EMD) that sees more than 110,000 patients per year. With the emergence of COVID-19, containment strategies were implemented nationally, requiring isolation of large numbers of patients and placing an extraordinary demand on the existing healthcare infrastructure. A multidisciplinary taskforce was assembled to plan for surge capacity, with the intent to create an “EMD extension,” a temporary outdoor facility to manage patients with suspected COVID-19 and relieve the pressure on the existing EMD isolation facilities.

**Fig. 1.**
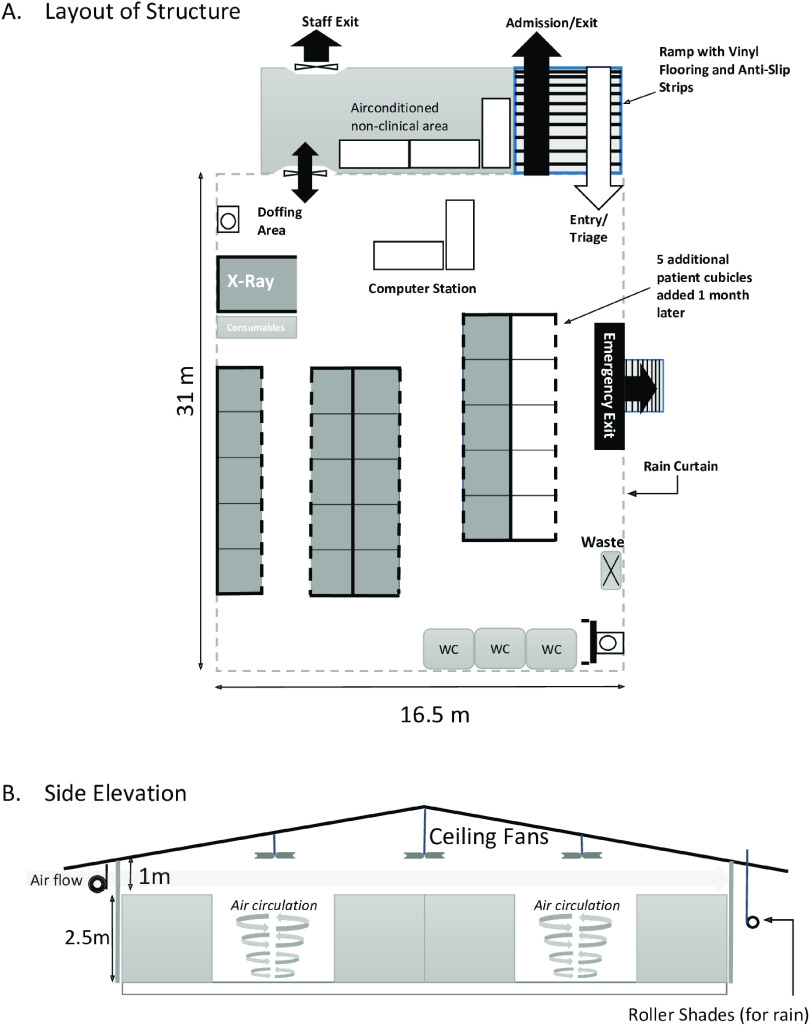
Layout and side elevation of emergency medicine department extension facility. Not drawn to scale.

We designed an A-shaped structure with open-air sides and a canvas roof, a perimeter fence for privacy, and ceiling fans to maximize air flow. The structure measured 31 m × 16.5 m, with a height of 5 m at its highest point (Fig. 1). This temporary structure was built on an existing empty flat concrete platform, which was covered with vinyl flooring. The structure contained a resuscitation bay and a radiology cubicle with walls lined with lead shields; an adjacent air-conditioned cabin served as a rest area for staff.

The structure has 20 patient cubicles (2 m × 2 m), constructed using aluminum framing and polyvinyl chloride (PVC) panels. The walls are 2.5 m high, with 3 fixed and 1 curtained side. The modular nature of the construction allowed for 5 additional cubicles to be added later.

Alcohol-based hand rub (ABHR) dispensers were placed in patient cubicles, at healthcare worker (HCW) stations, at medication preparation station, and in the donning and doffing areas. Hand hygiene sinks were installed and connected to the main water supply line, and portable toilets were connected to central sewage.

We engaged a local tent contractor without specific expertise in medical or emergency response structures, although the designs were verified with an engineer. The structure was erected within 17 hours, followed by fire safety checks, temperature monitoring, rehearsals of patient movement and resuscitation drills, and a smoke test that demonstrated good air flow. The structure was fully operational within 6 days.

Staff working in the extension building wear long-sleeved gowns, N95 masks, gloves and eye protection at all times, changing gloves and performing hand hygiene between patients. Donning and doffing areas for personal protective equipment (PPE) were designated near entry and exit points.

Between February 14, and May 12, 2020, the EMD extension received 5,004 patients, while 9,374 patients were seen in the main EMD isolation facility. In total, 710 confirmed COVID-19 cases were managed in the NUH EMD. No infection prevention and control (IPC) breaches or exposures were observed in the EMD extension. No transmission of COVID-19 to HCWs or patients has been identified thus far at NUH.

The maximum temperature in Singapore remains 31–33°C year-round. With the use of portable cooler units and shade cover, the average internal temperature was maintained at 28.1°C, with 90% of staff stating they could wear required PPE for >1 hour without discomfort.

This report demonstrates the potential for an adapted structure to provide rapid, safe and effective surge capacity for the triage, screening and management of COVID-19 patients. This design successfully incorporated commonly sourced building materials, and natural ventilation, as a rapid solution for the screening and care of COVID-19 patients. Here, we utilized existing plumbing and water lines, but portable alternatives could be substituted. Temperature control was managed effectively with portable coolers, and in colder climates portable heaters could be adapted.

Natural ventilation, while not commonly utilized in the west, remains a common design feature for hospital wards in other parts of the world. Although AII rooms with 12 air changes per hour (ACH) have become the gold standard for airborne diseases, natural ventilation may provide rates that exceed these requirements^8^ and could represent a low-cost alternative. ACH of up to 40 have been reported in naturally ventilated structures with high ceilings,^8^ and such designs were historically used in the construction of tuberculosis wards.^9^ In recently published guidelines on the design of severe acute respiratory infection (SARI) treatment centers in limited-resource settings, natural ventilation is highlighted as one potential solution.^10^

Literature describing the use of natural ventilation combined with low-cost and low-tech materials for the safe management of COVID-19 patients is scarce. This report, in conjunction with older studies recognizing the utility of natural ventilation, lends credence to the idea that low-cost, rapidly erected structures (ie, open-air tents, without HEPA filtration units) may be a solution to managing the surge of COVID-19 patients, particularly in low-income countries, or other areas with depleted medical capacity.
